# Idealism, materialism, and Vygotsky’s cultural historical theory

**DOI:** 10.3389/fpsyg.2024.1425751

**Published:** 2024-07-08

**Authors:** Gustav A. von Schulz

**Affiliations:** Law School, Yangzhou University, Yangzhou, China

**Keywords:** Vygotsky, Cultural Historical Theory, idealism, materialism, monism, dualism, hermeneutics

## Abstract

Vygotsky straddled the period of the Russian Revolution and found himself facing the Marxist materialist ideology of the Soviet Union with the need for a new method of psychology. Ironically, the Soviet Union’s need for a Marxist based method of psychology coincided with Vygotsky’s prior research on methods of interpretation which were inspired by Hegel and primarily based on the role of consciousness and culture. As a result of Vygotsky’s pre-revolutionary work and inspiration from Hegel clashing with the post-revolutionary need for a new methodology for psychology, Vygotsky developed his Cultural Historical Theory. In presenting his new theory, Vygotsky attempted to resolve a fundamental ideological conflict between idealism and materialism. Furthermore, Vygotsky worked to create an effective new research method by drawing inspiration from *Gestalt* psychology, Hegel, Marx, and Engels. The result of Vygotsky’s efforts was a theory based on psychology of consciousness and mind rather than a biology-based psychology focused entirely on analysis of stimuli and responses. In analyzing Vygotsky’s theory, it is useful to draw inspiration from Vygotsky’s criticism of pure empiricism, and to be inspired by Vygotsky’s demonstration on how deeply rooted differences between societies may be bridged by finding unexpected commonalities within opposing ideologies.

## Introduction

Vygotsky provided a unique perspective of understanding psychology through the culture and history of a people. This approach to the methodology of psychology may be reasonably seen as Vygotsky’s attempt to put on a Marxist veneer on ideas related to more theologically based topics such as hermeneutics and idealism. This conflict between Marxism and idealism reflects Vygotsky forming his perspectives through his life experience of moving from the society of Tsarist Russia and Vygotsky’s appreciation of Hegelian thought, towards the newly formed Soviet Union society based on Marxism. Notably, the Russian revolution occurred during the period of Vygotsky’s work on his dissertation, which would be published thirty years after his death as “Psychology of Art” (Vygotsky, 1965/1998). Furthermore, in Vygotsky’s works relating to his Cultural Historical Theory, he references his work on “Psychology of Art” despite this work being unpublished.

Many of the ideas for Vygotsky’s Cultural Historical Theory are provided in “Psychology of Art” and formed as the background to Vygotsky’s philosophy throughout his relatively short career. Vygotsky outlined his theory in terms of general psychology and used his new theory as the basis for understanding the development of human intelligence. Vygotsky presented his theory through the reconciliation between materialism and idealism which was at the core of the crisis in psychology occurring in the beginning of the 20th century. This crisis reflected the changes in worldview occurring after the Russian revolution and the fundamental shift in the guiding ideology of Vygotsky’s social environment.

After the Russian revolution, there was an apparent need in the Soviet Union to develop a new materialist theory of psychology based on Marxist historical materialism (Leontiev, 1982, p. 9). In that vein, Vygotsky emphasized that psychology needed its own version of Marx’s “Capital” which would serve as an illustration of psychology and demonstrate the materialistic dialectic in the form of psychological tools. This psychological “Capital” would allow the formation of a process from within psychology and discover facets of psychology that were beyond the reach of other methods of obtaining and organizing knowledge (Yaroshevsky and Gurgenidze, 1982a, p. 437). Vygotsky described his theory as “Cultural Historical” based on the psychological tools or system resources which are all created socially and are elements of culture (Leontiev, 1982, p. 27).

## Discussion

In 1925, Vygotsky published “Consciousness as a Problem of Psychological Behavior” within a collection of works titled “Psychology and Marxism” (Vygotsky, 1925/1982; Yaroshevsky and Gurgenidze, 1982b, p. 462). In this article, Vygotsky sought to provide a Marxist justification for the study of consciousness within psychology, despite the difficulty of reconciling the abstract nature of consciousness with materialism. To address this dilemma, Vygotsky began with a citation to Marx stating that even the worst human architect differs from the best insect because the architect, prior to building anything in reality, will first build it in his head (Vygotsky, 1925/1982, p. 78). Vygotsky further attributed to Marx the belief that a person not only changes the form of what is given by nature, but also actualizes his conscious goal. This actualization of a particular goal also determines the method and character of a person’s work which must be subordinate to that person’s will.

Vygotsky noted that the discipline of psychology avoided the problem of consciousness and, consequently, avoided researching the difficult problem of human behavior (Vygotsky, 1925/1982, pp. 78–83). In response, Vygotsky noted that psychology without consciousness creates a methodology which lacks the necessary methods of research of unapparent reactions, including internal movements and internal speech; furthermore, denial of consciousness results in biology consuming sociology, physiology, and psychology to the point that there are no principal boundaries between the behavior of animals and the behavior of humans. This circle of biological confusion also preserves the pre-existing dualism of spirituality of subjective psychology and permanently closes-off research in the area of the most important problems of human behavior and its forms, instead retaining a false understanding of behavior as a sum of reflexes. Vygotsky stated that instead of studying behavior as a reflex, behavior itself should be studied in terms of its mechanisms, contents, and structure. Likewise, consciousness should be in the same order as all the reactions of the human organism, and should not be looked at biologically, physiologically, or psychologically as a secondary occurrence.

Vygotsky emphasizes the fundamental distinction between humans and other animals. With respect to animals, Vygotsky sees two types of reactions: unconditioned and conditioned. However, Vygotsky believes that human behavior and human life is based on the broad experiences of one’s prior generations, but those experiences are not passed through birth, but are rather the historical experiences of one’s ancestry. According to Vygotsky, there are uniquely human social experiences which are the experiences of other people and are the “social component of human behavior” (Vygotsky, 1925/1982, pp. 83–84). A third experience affecting human behavior, which Vygotsky attributes to Marx instead of Hegel, is what Vygotsky called “duplicating experience”: the dual work of building something in one’s mind then building the same thing with one’s hands. This duplicating experience allows the development of active adaptation which is unavailable to other animals. Vygotsky supported this premise in his other writings by noting that lower animals normally function by trial and error through self-training without understanding their situation, yet Chimpanzees are different in that they can see the broader circumstance and engage in intellectual reaction through activity which appears counterproductive (Vygotsky, 1930/1982, p. 210).

In emphasizing the “duplicating experience” of human behavior, Vygotsky also considers the use of words and their role in regulating behavior as stimuli and reflex. A word which one hears acts as a stimulus, while a word which is said is a reflex, meaning that a reflex can become a stimulus and vice versa—this is the basis for social behavior and serves as a collective coordinator of behavior. From this premise and Hegel’s slave master dialectic (Hegel, 1807/2018, pp. 80–81), Vygotsky draws on speech as the basis of recognizing one’s self through the recognition of other psychologies (Vygotsky, 1925/1982, pp. 95–96). Namely, it is through the recognition of others, that we recognize ourselves since we are in a relationship with ourselves in the same way as others have a relationship with us: I only recognize “you” because I exist for myself as an “other.” Vygotsky draws this dialectic of one’s self-consciousness further to the division of personality between “I” and “it” which he attributes to Freud and the attribution to Lock that self-consciousness is the experiencing of what is happening in one’s own soul (Vygotsky, 1925/1982, pp. 90–96). Vygotsky further supports this reasoning by giving an example of the development of the consciousness of speech within the deaf and the partial development of tactile reaction in the blind.

One of the problems of the above reasoning along with the reasoning in “Psychology of Art” is that it strongly follows idealism in that it does not rely on experimental data or on anything which may be experimentally proven or disproven. This reliance on philosophy generated a degree of discomfort in the Soviet Union—a country which followed Marxism and the related ideology of materialism. Although Vygotsky joined in the advocacy to reform the sciences in the Soviet Union to make them conform with Marxism, Vygotsky was unwilling to let go of his methods of theoretical reasoning and instead attempted to redefine his reasoning as being consistent with Soviet Union’s materialist ideology.

The reconciliation of materialism and idealism was written about by Vygotsky in several articles, with “Historical Meaning of Psychological Crisis” and its method of psychological consciousness being a standout due to its detail, overtly religious tones, and not being published until 1982 (Editorial Board, 1982, p. iii). Due to “Historical Meaning of Psychological Crisis” being a more unrestrained view of Vygotsky’s ideas, it is first helpful to consider how his public writings reflected his beliefs prior and after the writing of “Historical Meaning of Psychological Crisis.”

One of the areas of interest for Vygotsky was Koffka’s *Gestalt* psychology and it was appropriate for him to provide a foreword to a 1926 Moscow publication of Koffka’s “Introspection and Method of Psychology.” In Vygotsky’s pre-word, he first praises the Soviet Union’s editorial team for looking beyond the Soviet Union and including a Western perspective in the collection volume titled “Problems of Modern Psychology” (Vygotsky, 1926/1982). Vygotsky stated that to build a system of Marxist psychology and resolve the crisis in psychology requires looking at a broad range of sources. Vygotsky also criticized the apparent perception that Marxists were reforming sciences for no apparent reason while in the West psychology was calm and it was as clear as physical sciences.

According to Vygotsky, the psychological crisis in Russia arose from the scholarly orientation on American behaviorism. Vygotsky regarded behaviorism as being helpful in moving psychology away from spiritualism and idealistic subjectivism; however, Vygotsky sought to create a different path from American behaviorism and Russian reflexology (Vygotsky, 1926/1982). Vygotsky presented a goal of building psychology as a science of behavior of a social person rather than as behavior of a high form mammal.

According to Vygotsky, *Gestalt* psychology included many of the elements which are necessary in Marxist psychology; however, there are areas of disagreement which are to be expected since *Gestalt* psychology was developed in the West, and is based on completely different philosophical roots, and originated in a completely different cultural environment (Vygotsky, 1926/1982, p. 102). Vygotsky praises *Gestalt* psychology for its materialistic monism and its methodology of descriptive introspective and objective reactive research (Vygotsky, 1926/1982, pp. 100–102). Vygotsky lists the negative side of *Gestalt* as the theory’s excessively close relationship between the problems of psychology and theoretical problems in theoretical physics and the lack of a social viewpoint in *Gestalt*’s “intuitive” theory of consciousness. While showing concern that in psychology’s movement towards materialism it still has a risk of being stuck in “idealism’s swamp,” Vygotsky observed that the arrival of *Gestalt* in the West signaled the development of psychological science in the same direction as Marxists reform of psychology.

In 1931, Vygotsky authored another foreword which appears to show his commitment to a view of psychology entirely grounded in materialism. He vehemently criticizes any consideration of spirit and focuses entirely on advocating a view of monistic materialism. Vygotsky directly criticizes as metaphysical the dualism in psychology as applied by Bergson in his concept of a dual memory of brain and spirit (Vygotsky, 1931/1982, pp. 151–155). According to the 1931 Vygotsky, Bergson’s theory reduces the brain to a tool which serves a mediating function and is based on idealism irrespective of whether the brain and spirit relationship is considered from a top-down or a bottom-up perspective. Namely, any linkage of psychology to the development of spirit presents psychological problems as metaphysical. Vygotsky observes this problem with some German scholars whose criticism of separation of psychology and philosophy relates to each professor having their own psychology. Vygotsky objects to those German scholars since they are implying that psychology does not exist as a precise science.

Vygotsky further criticizes that idealistic psychology is engaged in isolated studying of an independent kingdom of spirit without any relationship to the materialistic existence of a person (Vygotsky, 1931/1982, pp. 151–155). For example, metaphysical assumptions about psychology of memory leave pedagogy of memory without a psychological basis, and only a new viewpoint which uncovers psychology of memory from the perspective of its development can lead to a truly scientific formation of pedagogy of memory with psychology as its base.

In contrast to the above published articles, a 1927 article titled “Historical Meaning of Psychological Crisis” (Vygotsky, 1927/1982), which, while borrowing some ideas from the 1926 forward to Koffka’s book, provided much greater detail on Vygotsky’s thinking. In this 1927 article, Vygotsky holds a significantly more casual and sincere tone. “Historical Meaning of Psychological Crisis” reads as Vygotsky taking the role of a teacher who is conveying his sincere thoughts to his students, as compared to the published works which have a more declarative tone and are absolute in their devotion to materialism and Marxism. This difference in tone is immediately evident as Vygotsky opens “Historical Meaning of Psychological Crisis” with a quote from the bible which states that the stone which was despised by the builders is to become the cornerstone (“*Камень, который презрели строители, стал во главу угла…*”) (Vygotsky, 1927/1982, p. 291). The commentaries reference the book of Matthew from the Bible and attribute the Vygotsky’s “stone” to be the union of practice and philosophy in one (Yaroshevsky and Gurgenidze, 1982b, p. 468).

In reconciling Vygotsky’s devotion to idealism and the apparent embrace of dualism in “Historical Meaning of Psychological Crisis,” Vygotsky explains that, according to Engels’s worldview, ideas cannot just exist in themselves; rather, materialism is important because ideas are gathered around either idealism or materialism which correspond to the two poles of social life and the two primary struggling classes (Vygotsky, 1927/1982, p. 305). Thereby, Vygotsky reduces the difference between materialism and idealism as being a political difference rather than a substantial difference in scientific worldview. Vygotsky further states that since ideas in their role as philosophical facts have a more apparent social nature than scientific facts, the role of ideas ends as a covert ideologic agent disguised as scientific fact—the individual idea then dissolves and adds up in the class struggle of ideas. After making this broad statement that materialism and idealism are based on an ideological perception of ideas, Vygotsky draws broader conclusions on how general science studies material rather than existing purely on understanding and logic (Vygotsky, 1927/1982, p. 311).

Vygotsky argues that every natural scientific understanding arises from empirical facts and vice versa. No matter how abstract and removed an understanding, there is always some substance or residual of concrete reality from which the understanding arose. Therefore, reality exists even in the abstraction of mathematics; and, for this premise, Vygotsky cites examples from Marx and Engels (Vygotsky, 1927/1982, p. 311). Vygotsky notes that the reverse relationship between natural understanding and empirical facts also exists. Namely, that the most direct, empirical, raw, and unitary facts which are based in reality— already include the beginning of abstraction; therefore, material science is not the raw material from nature any more than general science is pure understanding (Vygotsky, 1927/1982, pp. 312–313).

According to Vygotsky, analysis is always a part of research, otherwise initial experiences would become merely registrations (Vygotsky, 1927/1982, p. 408). Therefore, the difference between general and empirical sciences is one of degree rather than substance. In other words, general physics remains physics rather than logic despite dealing with issues which are counter to observable reality. Vygotsky distinguishes general from specific science by analogy of using a microscope on a water slide for the purpose of studying water versus for the purpose of studying the functioning of the microscope itself (Vygotsky, 1927/1982, pp. 314–315). This conception of a reciprocal relationship between observed and theoretical knowledge also serves as the basis of Vygotsky’s theory of education.

According to Vygotsky, new research in general sciences does not obtain new forms of relationship in understanding, but rather new broad facts, such as evolution. Likewise, it is improper to divide material and its processing because understanding requires both. In that vein, Vygotsky defines general science as a science receiving material from individual sciences and performing the further processing and acculturation of material which would be impossible to perform within the individual sciences. Consequently, general science arises from the inability to further process material within individual sciences and relates to the theories, laws, hypotheses, and methods of individual sciences in the same way as individual sciences relate to the facts of reality which they study (Vygotsky, 1927/1982, pp. 317–320). Vygotsky cites Engels and argues that even Hegel’s classification system is one based on the laws of nature rather than entirely based on thought. With this reasoning, Vygotsky states that general psychology is a part of the Hegelian dialectic. Vygotsky then concludes that a general principle of general psychology is the link between thinking and existence in science which is simultaneously an object, a higher level of criterion, and a method (Vygotsky, 1927/1982, pp. 322–323).

Scientific knowledge differentiates between the analysis of facts and analysis of understandings. Every naming of an object is a theory since naming attaches understanding, and every naming is a critique of a word which broadens its meaning (Vygotsky, 1968/1982, p. 164). In other words, “to say” is “to give a theory” and a world of objects occurs where there arises a world of names (Vygotsky, 1968/1982, p. 164). If it was otherwise, then science would merely discover facts without expanding understanding and there would be no new discoveries, but rather new examples of existing understandings (Vygotsky, 1927/1982, p. 316). With every word being a theory, a child naming objects is already forming a process of discovery through the act of classification. Language itself is imbedded with the possibility of a scientific recognition of fact—a word is already a fetus of science, and, in this sense, it can be said that in the beginning of science there was word (“*Слово и есть зародыш науки, и в этом смысле можно сказать, что в начале науки было слово.*”) (Vygotsky, 1927/1982, pp. 313–314).

Vygotsky manipulates Russian grammar in an interesting manner where his use of “word” is referencing the “Word,” as in the “Word of God,” but also referencing a “word” in language. The use of “Word” in the beginning of sentences takes advantage of Russian grammar’s liberal word order rules and creates further ambiguity with capitalization of “Word” when referencing “God” versus non-capitalization of a human “word.” The ambiguity is effective because in the Russian language there are also no definite or indefinite articles such as “a” or “the.” However, the use of the singular “*slovo*” instead of the plural “*slova*,” combined with the biblical reference at the beginning of the book and the biblical reference of “in the beginning there was Word” distinctly points to a religious implication to Vygotsky’s use of both “Word” and “word.” Particularly when considering the book of John in the Bible: (“In the beginning was the Word, and the Word was with God, and the Word was God.”) (King James Bible, 1769/2024, John 1:1). The grammatical play on words and linking the Word to a word is evident, albeit less explicit than Gadamer’s comparison between human words and the divine “Word” (Gadamer, 2006, pp. 419–423). In effect, Vygotsky is stating that God was at the creation of science, and science begins with human language: namely, science’s origin is tied both to God and to people.

Vygotsky continued to push towards eliminating the distinction between materialism and idealism by arguing that a “genius” could see that Hegel’s system of idealism rested on the head of materialism (Vygotsky, 1927/1982, p. 336). Namely, Hegel was “limping” towards the truth because his dialectics are methodological truths that are separated from factual lies. However, there is some evident irony of Vygotsky using the word “genius,” despite Hegel’s extensive critique this term (Hegel, 1807/2018, pp. 31–32)—this irony may be Vygotsky indirectly self-criticizing his own attempt to see Hegel as a materialist.

Vygotsky observed that, broadly speaking, there exist two psychologies: a natural-scientific materialistic psychology and a spiritual psychology (Vygotsky, 1927/1982, pp. 381–383). Vygotsky observed that the psychology of his time was based on the study of the soul without a soul which leads to: First, the problem of descriptive psychology which describes and understands rather than explains. Second, a descriptive natural psychology which constructs a determinative criminal law, leaves no space for freedom, and does not resolve the problem of culture. Vygotsky also criticizes analytical psychology as non-scientific because the gap between psychology and physics is too great to allow for understanding the relationship between the two fields. According to Vygotsky, the very nature of psychological material does not permit separation of psychological position from philosophical theories to the extent which can be found in other sciences (Vygotsky, 1927/1982, p. 391). Finally, Vygotsky, citing Munsterberg, explains that fundamental problems of psychology ultimately belong to philosophy and psychologists fall into self-deception when they imagine their laboratory work could possibly lead to solutions of those fundamental problems.

Vygotsky explains that science can study that which is not given directly, such as children’s consciousness, by creating an object of study through a method of interpretation of traces or effects (Vygotsky, 1927/1982, pp. 343–346). This is analogous to using a thermometer for analyzing increase in temperature by reconstructing the object of study (the temperature) through the reconstruction of subject of study (the rise in mercury); however, this type of study of the temperature is different from the study of the physical process of rising mercury. Children’s psychology likewise leaves tracks and perhaps those tracks which are left in adults can be interpreted analogously to reading a thermometer for interpreting increases in temperature.

Vygotsky refers to his writings on psychology of art and accentuates his assumption that developed forms of art provide a key to the undeveloped forms rather than in reverse: namely, Vygotsky believes that Shakespeare gives clues on primal art rather than the reverse (Vygotsky, 1927/1982, p. 405). Using this as his basis, Vygotsky builds a foundation on forming a Marxist methodology of psychology. Ironically, the idea of developed forms incorporating and destroying, or “sublating,” earlier forms is closely tied to Hegel’s “Phenomenology of Spirit” (Hegel, 1807/2018).

Vygotsky reframes his writings on psychology of art [this is a reference is to Vygotsky’s unpublished manuscript, which was published 30 years after his death as “Psychology of Art” (Vygotsky, 1965/1998)] as being focused on the process rather than the substance of “analysis of aesthetic reaction” (Vygotsky, 1927/1982, pp. 405–408). The single parable, novella, and tragedy in “Psychology of Art” are not discussed in detail and are intentionally ugly examples of those genres to emphasize the basic elements of those genres and to demonstrate that an objective-analytical method is similar to an experimental method. The significance of the objective-analytical method is wider than its specific area of observation; consequently, every poem or example is an experiment, and the goal of its analysis is to reveal the substantive principle or law within the given natural experiment. Therefore, the objective-analytical method is a method of real natural science, as compared to a phenomenological method which, in the first place, is a method of mathematics and pure sciences of the spirit. In further advocating for the objective-analytical method, Vygotsky states that Marx’s “Capital” was written using this objective-analytical method because Marx analyzes market prices as merely a single cell of bourgeois society, and, through this method, Marx demonstrates that the development of the entire body (the bourgeois society) is easier to study than the cell. Therefore, true social psychology starts from Marx’s “Capital” (Vygotsky, 1927/1982, pp. 421–422).

The above discussion focuses on Vygotsky’s perception of method of psychology and provides a particular insight into Vygotsky’s philosophical thinking during his life. Furthermore, there appeared to be a dualism in Vygotsky’s thinking with certain writings remaining unpublished during his lifetime and other writings being published immediately after they were written. In effect, when Vygotsky argues for a narrow, ideologically materialistic viewpoint, he is publishing his works; however, when Vygotsky attempts to wade into idealism, religion, and Hegel, his works do not get published until decades after his death. Although some of the philosophical, methodological, and theoretical works of Vygotsky were unpublished, there is no doubt they were still utilized since the ideas in those works were passed to Vygotsky’s countless students and in his communications with other scholars (Yaroshevsky and Gurgenidze, 1982a, p. 455).

## Conclusion and implications

Vygotsky presents his Cultural Historical Theory as a Marxist method of psychology inspired by Marx’s “Capital”; however, the substance of Vygotsky’s proposed method is much closer Hegel’s idealism than the materialist ideology of the Soviet Union. Many of the ideas expressed in Vygotsky’s “Psychology of Art” can be traced back to Hegel’s discussion of the *Geist* in the form of reason and the related production of an ideal work and its reduction to physical labor (Hegel, 1807/2018, pp. 95–97). Ironically, Vygotsky attributes this fundamental concept to Marx rather than Hegel by attributing to Marx a quote regarding the uniquely human character of building a plan in one’s mind prior to building anything in actuality.

Beyond Vygotsky attributing Hegel’s ideas to Marx, Vygotsky references Engels to reduce any philosophical or theoretical differences between materialism and idealism to mere political rivalry between the Soviet Union and Western countries. Vygotsky also appears to embrace some aspects of theology which was likewise at tension with the atheist ideology of the Soviet Union. In this respect, it is worthwhile reflecting on how Vygotsky’s presentation of a methodology based on cultural and historical understanding of Marxism has very close parallels to the hermeneutic methodology presented by Gadamer at approximately the same time as “Psychology of Art” was first published in the Soviet Union.

Overall, irrespective of the ideological debates underlying Vygotsky’s Cultural Historical Theory, this theory provides a meaningful methodology for considering psychological phenomena beyond pure experimental study. Vygotsky properly observed that experimental work on stimuli and reaction is insufficient to study the more comprehensive psychological experience of humans. Furthermore, the nature of human behavior is such that it cannot be fully reduced to experimental study without reducing the scope of study to such a degree that the results are either trivial or anecdotal in their substance. Consequently, Vygotsky’s ideas continue to be relevant in contemporary times where there continues to be a need for theoretical research in psychology to provide a basis for experimental research and to prevent empirical research from being reduced to a method of “trial and error.”

## Author contributions

GvS: Writing – original draft, Writing – review & editing.
